# Inflammatory myofibroblastic tumor of maxillary sinus successfully treated with radiotherapy and corticosteroid: report of a rare case

**DOI:** 10.1186/s43046-020-00038-0

**Published:** 2020-06-01

**Authors:** Rituparna Biswas, Anirban Halder, Mimi Gangopadhyay, Dipanwita Biswas

**Affiliations:** 1 Department of Radiation Oncology, Medica Cancer Hospital, Rangapani, Siliguri, India; 2 Department of Pathology, Medica North Bengal Clinic, Siliguri, India; 3 R G Kar Medical College, Kolkata, India

**Keywords:** Inflammatory myofibroblastic tumor, Maxillary sinus, Prednisolone

## Abstract

**Background:**

Inflammatory myofibroblastic tumor (IMT) is a mesenchymal tumor mainly found in lung or retroperitoneum and rarely affects head and neck region. Extensive English literature search reveals that less than fifty cases of head and neck IMT have been reported so far, maxillary sinus being fewer.

**Case presentation:**

We present a case of IMT involving maxillary sinus in a 48-year-old gentleman who attained complete clinico-radiologic response after treatment with radiotherapy (RT) and concurrent oral prednisolone.

**Conclusions:**

This is the first report where such magnificent response was attained in primary setting treated with RT and steroids as opposed to surgery which used to be considered as standard of care till now.

## Background

Inflammatory myofibroblastic tumor (IMT) is a rare entity with controversial biological behavior. It is now considered as a benign neoplasm with locally aggressive behavior, having rare metastatic potential [1]. The World Health Organization (WHO) defined IMT as an intermediate soft tissue tumor that is composed of myofibroblastic and fibroblastic spindle cells accompanied by infiltrate of numerous inflammatory cells, plasma cells, eosinophils, and lymphocytes [1]. IMTs are usually found in the lung, abdomen, retroperitoneum, and extremities, rarely affecting head and neck region [2]. Whether IMT is a neoplastic or a reactive process had been a matter of controversy, but identification of anaplastic lymphoma kinase (ALK) gene rearrangement suggests more of a neoplastic etiology than a reactive inflammatory process [3]. The exact etiology and pathogenesis are not clearly understood due to paucity of cases; however, reports indicating infection or an abnormal immunological reaction are considered as possibilities [1, 4]. Similarly, no standardized treatment guidelines are available yet. Herein, we present a case of IMT involving maxillary sinus in a 48-year-old gentleman who attained complete response after treatment with radiotherapy and oral prednisolone.

## Case presentation

**Fig. 1:**
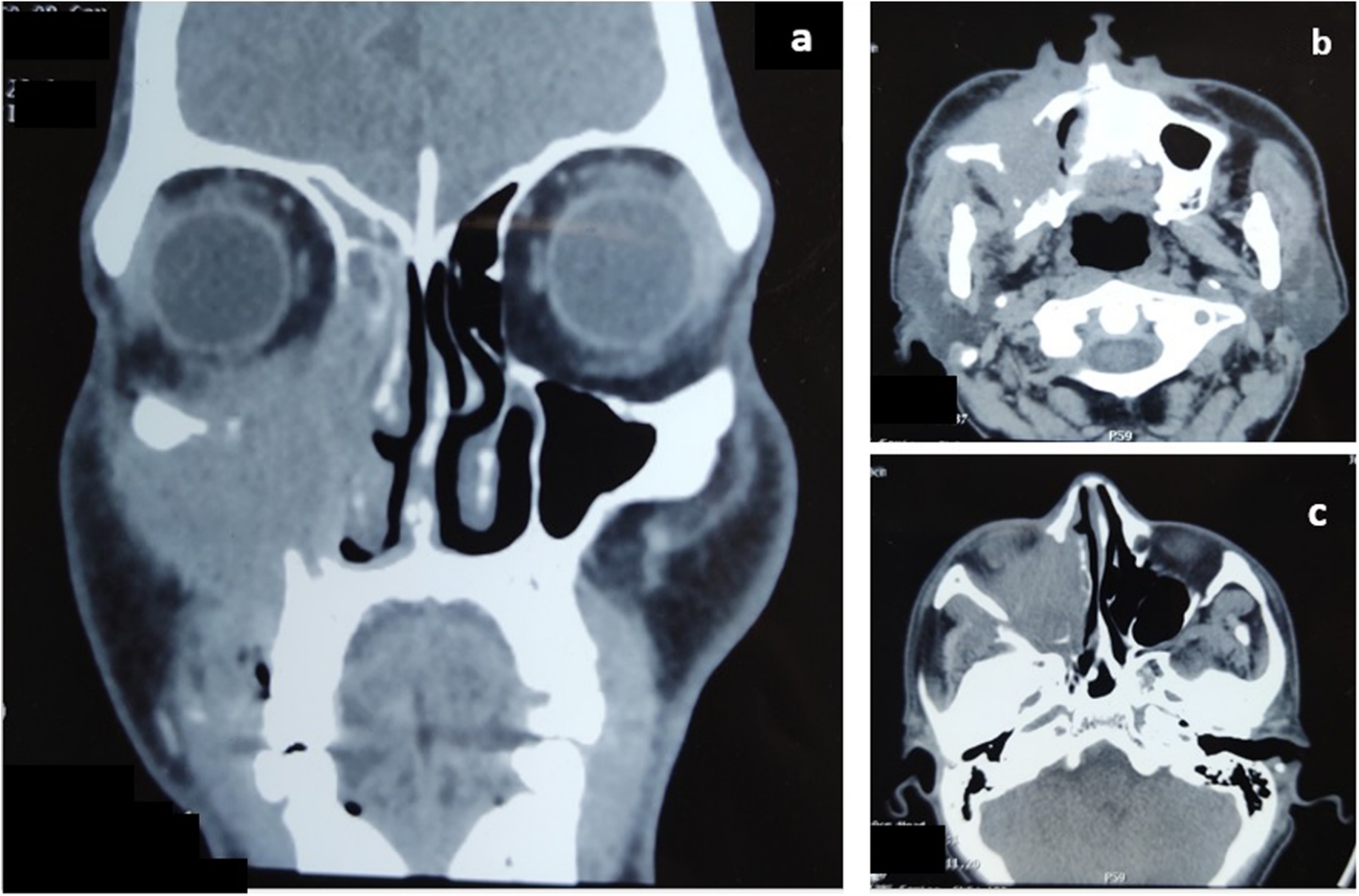
CECT scan showing right maxillary tumor with **a** extension to orbit (sagittal view), **b** anterior and posterolateral extension (axial view), and **c** meatus involvement and intraorbital invasion (axial view)

**Fig. 2:**
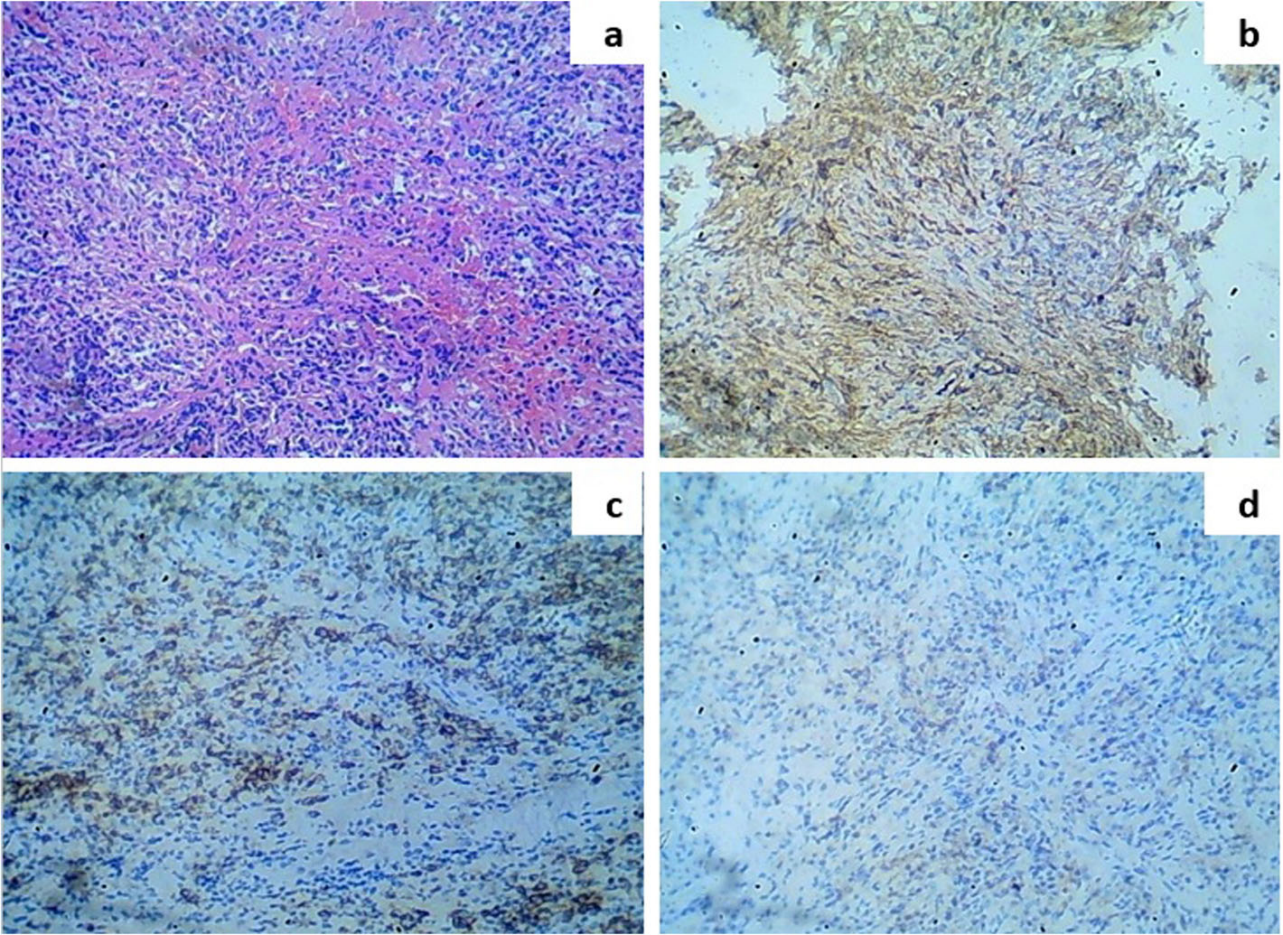
**a** Hematoxylin and eosin staining, **b** cells staining with lambda, **c** CD138 staining, and **d** cells positive for CD19

An otherwise well, a 48-year-old male patient presented with pain and swelling over right maxillary region for 4 months associated with inability to open mouth fully. Clinically, he was having fullness over right maxillary region and severe trismus (15 mm oral opening). Contrast-enhanced computed tomography (CECT) scan of face and neck region revealed a soft tissue mass in right maxillary antrum with bony destructions—intraorbital extraconal extension abutting the inferior rectus muscle with possible involvement of the infraorbital nerve, medial extension to middle meatus, inferior meatus and involving nasolacrimal duct, anterior extension to premaxillary space, inferior extension to alveolar process and posterolateral extension to infratemporal fossa, and pterygoid fossa with possible involvement of temporalis and lateral pterygoid muscles (Fig. 1). No evidence of lymph nodal or distant metastases was found on imaging. Biopsy from tumor was taken for histopathologic examination (HPE). HPE and subsequent immunohistochemistry (IHC) suggested it to be inflammatory myofibroblastic tumor (Fig. 2). Morphologically tissue consisted of plasma cells, eosinophils, and lymphocytes in a variably collagenized spindle cell stroma. The spindle cells had bland nuclear morphology. On IHC, tumor cells were positive for CD3, CD20, CD 138, CD19, kappa, lambda (kappa:lambda was 2:1), SMA, and calponin and negative for CD56, CK, Alk-1, desmin, h-Caldesmon, and Ki-67 was < 1%.

**Fig. 3:**
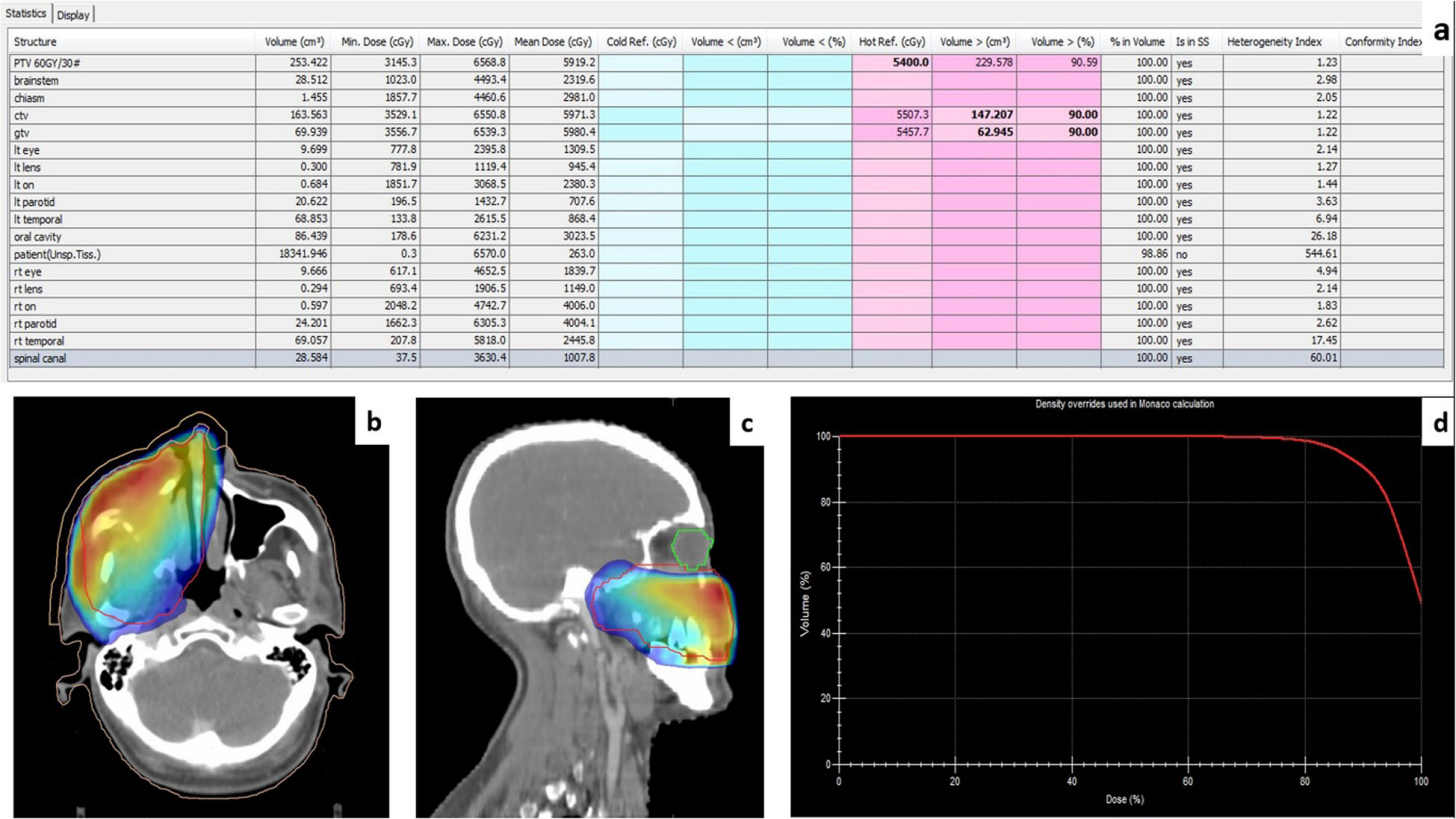
**a** Table showing dose received by OAR. **b** and **c** IMRT plan showing dose color wash of 90% isodose coverage of PTV. **d** DVH of RT plan (red line = PTV)

**Fig. 4:**
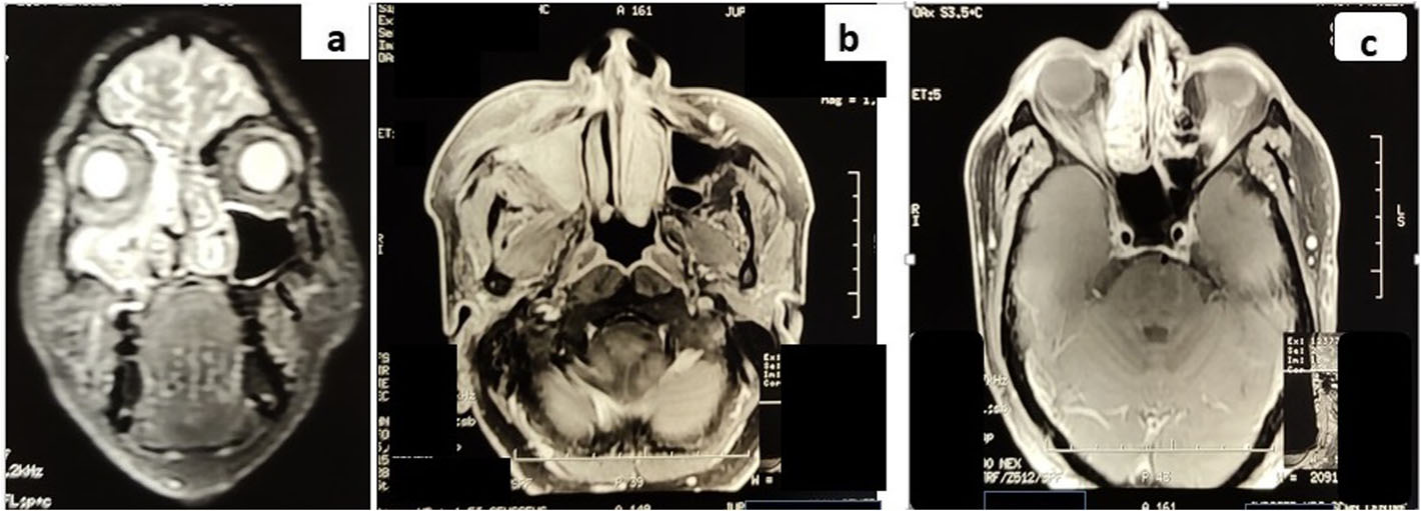
CEMRI showing post RT changes in **a** sagittal view and **b**, **c** axial view showing diffuse mucosal thickening without any evidence of enhancing solid tumor

In view of extensive disease, he was deemed unresectable. Hence, he was treated with definitive radiotherapy (RT) with dose 60 Gy in conventional fractionation over 6 weeks concurrent with oral steroids. For simulation and treatment, he was immobilized with thermoplastic mold in supine position with head extended using head rest. Intraoral stent was inserted to depress tongue outside radiation field as much as possible. CT simulation using 3 mm thickness with IV contrast was performed. Wet cotton bolus was used during treatment. For target volume delineation, all visible tumor in CECT scan was taken into account as gross tumor volume (GTV); for clinical target volume (CTV), 1 cm margin was added to GTV all around and then carved out respecting anatomical boundaries. Ipsilateral nasal cavity medially till nasal septum, entire maxillary sinus, anteriorly and laterally till skin, ipsilateral ethmoid sinus, posteriorly infratemporal fossa, and pterygopalatine fossa were encompassed inside CTV. Superiorly intraorbital margins of CTV were reduced to exclude eye. Finally, 5-mm margin to CTV was given to produce planning target volume (PTV); however, margin was reduced to 1 mm beside ipsilateral eye and optic nerve. Radiation planning was done by forward planning IMRT (intensity-modulated radiation therapy) (Fig. 3). Prednisolone was given in tapering doses with starting dose 60 mg and was tapered by 10 mg every subsequent week. He responded well. After 1 month of RT completion, clinically no swelling was appreciated and trismus resolved. Contrast-enhanced magnetic resonance imaging (CEMRI) was done after 2 months of RT completion which revealed post-RT changes with complete resolution of tumor (Fig. 4). He is doing fine till 6 months follow-up now.

## Discussion

As IMT of the maxillary sinus is a rare entity, knowledge pertaining to this is availed from case reports and series described in English literature. It usually presents as local pain or swelling as is found in index case. Unlike IMTs at other locations, systemic manifestations such as anorexia, weight loss, and fever have not been observed in cases of the maxillary sinus IMTs [5]. CT scan or MRI of IMTs in the paranasal sinus often suggests infiltrative growth with aggressive malignant potential [5]. It usually appears as homogeneous soft tissue masses filling the maxillary sinuses with no evidence of calcification or central necrosis. Bone destruction is frequently found in advanced cases as also evident in our case. Histologically, IMT is composed of variable admixture of fascicles of myofibroblastic spindle cells with a prominent polyclonal infiltrate of numerous plasma cells, lymphocytes, and acute inflammatory cells, in a loose myxoid or edematous stroma [5]. IHC is considered gold standard in obtaining diagnosis. Till date, many cellular markers have been identified including desmin, vimentin, smooth muscle actin, cytokeratin, and ALK-1 that aid in the pathologic diagnosis of IMT [2, 6]. As of now, surgical resection with negative margins is considered mainstay of management [2, 6, 7]. Where partial resection was only feasible, adjuvant radiotherapy or steroids were employed with promising results. In recurrent or refractory cases, radiotherapy and pharmacotherapies such as NSAIDs, COX inhibitors, corticosteroids, and kinase inhibitors were used alone or in combination [7]. Reports in favor of chemotherapy are scarce [8]. To date, no standardized chemotherapeutic regimens had been introduced. Radiotherapy in dose range 20–60 Gy has been administered in various reports [5, 6]. Our presented case attained complete clinico-radiological response with radiation therapy (RT) dose 60 Gy along with concurrent oral high-dose prednisolone in tapering dose. Extensive literature search reveals this is the first report where such overwhelming response was attained in primary setting treated with RT and steroids. Hence, our report suggests that RT with steroids is equally effective alternative to surgery in treating a patient with maxillary IMT. However, further studies with large sample size will be needed to corroborate our finding.

## Conclusion

We report the first known case of IMT of maxillary sinus being treated by radiotherapy and steroid who attained complete response, hence leading to advancement in management and care of individuals affected by IMT. Our evidence establishes radiotherapy with steroid as alternative to surgery which used to be considered as standard of care till now and this paves way for future studies.

## Data Availability

Not applicable

## Abbreviations

IMT: Inflammatory myofibroblastic tumor
RT: Radiotherapy
ALK: Anaplastic lymphoma kinase
CECT: Contrast-enhanced computed tomography
HPE: Histopathologic examination
IHC: Immunohistochemistry
IMRT: Intensity-modulated radiation therapy
DVH: Dose-volume histogram
OAR: Organs at risk
CEMRI: Contrast-enhanced magnetic resonance imaging

## References

[1] Coffin CM, Hornick JL, Fletcher CD. Inflammatory myofibroblastic tumor: comparison of clinicopathologic, histologic, and immunohistochemical features including ALK expression in atypical and aggressive cases. Am J Surg Pathol. 2007;31:509–20. 17414097 10.1097/01.pas.0000213393.57322.c7

[2] Salehinejad J, Pazouki M, Gerayeli MA. Malignant inflammatory myofibroblastic tumor of the maxillary sinus. J Oral Maxillofac Pathol. 2013;17:306–10. 24250100 10.4103/0973-029X.119754 PMC3830248

[3] Griffin CA, Hawkins AL, Dvorak C, Henkle C, Ellingham T, Perlman EJ. Recurrent involvement of 2p23 in inflammatory myofibroblastic tumors. Cancer Research. 1999;59(12):2776–80. 10383129

[4] Ushio M, Takeuchi N, Kikuchi S, Kaga K. Inflammatory pseudotumor of the paranasal sinuses: a case report. Auris Nasus Larynx. 2007;34:533–6. 17331689 10.1016/j.anl.2007.01.003

[5] Gale N, Zidar N, Podboj J, Volavšek M, Luzar B. Inflammatory myofibroblastic tumour of paranasal sinuses withfatal outcome: reactive lesion or tumour? J Clin Pathol. 2003;56(9):715–7. 12944561 10.1136/jcp.56.9.715 PMC1770050

[6] Hansen CC, Eisenbach C, Torres C, Graham S, Hardwicke F. Maxillary sinus inflammatory myofibroblastic tumors: a review and case report. Case Rep Oncol Med. 2015. 10.1155/2015/953857. 10.1155/2015/953857 PMC4339865 25763286

[7] Zhu Z, Zha Y, Wang W, Wang X, Gao Y, Lv W. Inflammatory myofibroblastic tumors in paranasal sinus and nasopharynx: a clinical retrospective study of 13 cases. BioMed Res Int. 2018. 10.1155/2018/7928241. 10.1155/2018/7928241 PMC6205320 30410939

[8] Al-Sindi K, Al-Shehabi MH, Al-Khalifa SA. Inflammatory myofibroblastic tumor of paranasal sinuses. Saudi Med J. 2007;28:623–7. 17457491

